# Immunotherapy with Native Molecule rather than Hypoallergenic Variant of Pru p 3, the Major Peach Allergen, Shows Beneficial Effects in Mice

**DOI:** 10.1155/2018/3479185

**Published:** 2018-06-13

**Authors:** Maria J. Rodriguez, Andrea Wangorsch, Francisca Gomez, Stefan Schülke, Maria J. Torres, Stefan Vieths, Stephan Scheurer, Masako Toda, Cristobalina Mayorga

**Affiliations:** ^1^Research Laboratory, IBIMA-Hospital Regional Universitario de Malaga-UMA, Pabellón 5 Sótano, 29009 Malaga, Spain; ^2^Molecular Allergology, Paul-Ehrlich-Institut, Paul Ehrlich Street 51-59, 63225 Langen, Germany; ^3^Allergy Service, IBIMA-Hospital Regional Universitario de Malaga-UMA, Malaga, Spain

## Abstract

**Background:**

The use of hypoallergenic derivatives is considered beneficial to promote the safety and efficacy of allergen-specific immunotherapy. We aimed to assess the efficacy of reduced and alkylated (R/A) Pru p 3, a hypoallergenic folding variant of the major peach allergen, in subcutaneous immunotherapy (SCIT) using a murine model of peach allergy.

**Methods and Results:**

After sensitization with Pru p 3, BALB/c mice received SCIT with Pru p 3 or R/A Pru p 3 and were challenged with Pru p 3. SCIT with Pru p 3, but not with R/A Pru p 3, suppressed anaphylaxis upon the challenge significantly. SCIT with Pru p 3 did not suppress Pru p 3-specific IgE and IgG1 production, but enhanced IgG2a production. In contrast, SCIT with R/A Pru p 3 suppressed IgE and IgG1 production, but enhanced IgG2a production only moderately. The therapeutic efficacy of SCIT with Pru p 3 was associated with induction of IL-10 and IFN-*γ*.

**Conclusion:**

Hypoallergenic folding variant of Pru p 3 is not likely an efficacious therapeutic component in SCIT of peach allergy. The lower efficacy of R/A Pru p 3 might be attributed to poor antigenicity and/or weak stability due to its unfolded conformation.

## 1. Introduction

The prevalence of food allergy has increased over the past decade. Allergen-specific immunotherapy (AIT) is the specific and disease-modifying approach to treat allergic diseases [1, 2]. However, allergen products for AIT have obtained marketing authorization only for certain respiratory allergies, for example, grass pollen allergy and house dust mite allergy as well as insect venom allergies, but not for food allergy due to unfavorable risk-to-benefit ratios which were observed in early clinical trials in peanut allergy [3]. Current AIT with native allergen extracts carries the risk of adverse reactions, since allergens in their native conformation present IgE epitopes and possess full allergenic potential. The risk of adverse reactions is concerned particularly in subcutaneous immunotherapy (SCIT), which is based on repeated injections of crude allergen extracts. To reduce the risk of adverse reactions, hypoallergenic variants with reduced IgE reactivity but with retained T-cell epitopes have been considered as safer and potentially more efficacious alternatives to the corresponding wild-type allergens [1, 4–6].

Pru p 3 is the major allergen for peach-allergic patients in the Mediterranean area and belongs to the nonspecific lipid transfer protein (nsLTP) family [7]. Primary sensitization to Pru p 3 and subsequent IgE cross-reactivity with the other members of the nsLTP family is considered to be responsible for manifestation of clinical cross-reactivity in food and certain pollen allergies [8, 9]. nsLTPs are characterized by an all-*α*-type compact and small structure with 4 *α*-helices and stabilized by four highly conserved intramolecular disulphide bonds [8]. Therefore, nsLTPs display high stability to thermal processing and gastrointestinal digestion, probably contributing to the frequently observed manifestation of systemic and severe symptoms [10]. In the previous study, we showed that a hypoallergenic folding variant of Pru p 3 can be generated by disruption of disulphide bonds upon reduction and alkylation [11]. Reduced and alkylated (R/A) Pru p 3 significantly diminished antigenicity and allergenicity, but retained T-cell immunogenicity [11].

Evidence has accumulated that the efficacy of AIT could be associated with induction of regulatory T-cells and/or immune deviation to a balanced Th1/Th2 type [12]. Since R/A Pru p 3 retained T-cell immunogenicity, we hypothesized that it induces the desired T-cell regulation in AIT to treat peach allergy. To verify the hypothesis, in the present study, we compared the efficacy of Pru p 3 and R/A Pru p 3 as a therapeutic component in SCIT to treat peach allergy using a murine model. Unexpectedly, although R/A Pru p 3 was able to reduce Pru p 3-specific Th2 responses, only a high dose of Pru p 3, but not R/A Pru p 3, suppressed peach allergen-induced anaphylaxis in the immunized mice significantly.

## 2. Methods

### 2.1. Allergen Preparation

Pru p 3 was purified from freshly prepared peach peel extract as previously described [11]. To disrupt the tertiary structure of Pru p 3, the allergen was denatured in 5.3 mol/L urea (in 0.13 mol/L Tris-HCl, pH 9.0), reduced by 28 mmol/L dithiothreitol at 55°C for 45 minutes, and alkylated by adding iodoacetamide to the final concentration of 55.5 mmol/L at room temperature. R/A Pru p 3 was then dialyzed against phosphate-buffered saline (PBS). For immunization and immunological analysis, freshly prepared Pru p 3 and R/A Pru p 3 were applied. For assessment for stability of Pru p 3 and R/A Pru p 3 during storage, sample solutions were kept at 4°C for 14 months. Protein concentrations were determined by Pierce BCA Protein Assay Kit (Thermo Fisher Scientific, Bonn, Germany). Endotoxin concentrations in the Pru p 3 samples, determined via chromogenic *Limulus* amebocyte lysate (LAL) test (Charles River PTS LAL), were less than 0.12 pg/10 *μ*g protein.

### 2.2. Electrophoresis and Immunoblotting

Native and R/A Pru p 3 were applied to SDS-PAGE (16%, 5 *μ*g/slot for Coomassie staining and 2.0 *μ*g/slot for immunoblotting) under nonreducing or reducing conditions with 1 mM dithiothreitol, and stained with GelCode™ Blue Safe Protein Stain (Fisher Scientific, Schwerte, Germany). For immunoblotting, proteins were transferred to a nitrocellulose membrane (BA85, 0.2 *μ*m, Schleicher and Schüll, Dassel, Germany) by semidry blotting, followed by Ponceau S staining (Merck, Darmstadt, Germany) of transferred proteins. Membranes were blocked in Tris-buffered saline containing 0.3% Tween 20 and incubated with a patients' serum (1 : 10), followed by mouse anti-human IgE coupled to alkaline phosphatase (BD Biosciences, Heidelberg, Germany) as secondary antibody. Bound antibodies were visualized with nitroblue tetrazolium/5-bromo-4-chloro-3-indolyl phosphate (NBT/BCIP) as substrate (Bio-Rad, Munich, Germany).

### 2.3. Immunization of Mice

The efficacy of SCIT was assessed using a murine model of peach allergy, which we established previously [13, 14]. BALB/c mice (female, 8–10 weeks: Charles River Laboratories Int.) were anaesthetized with inhaled sevoflurane and sensitized intranasally with 20 *μ*g of Pru p 3 plus 20 ng of lipopolysaccharide (InvivoGen, San Diego, CA) as an adjuvant. This immunization route was chosen because of the capacity of Pru p 3 to sensitize individuals through inhalation and in order to minimize the dose of protein needed [13–15].

Following a protocol [14], administration schedule was of three consecutive days plus once a week for four weeks. One week after the final sensitization (day 36), mice received subcutaneous immunotherapy (SCIT) with either 10 or 100 *μ*g of Pru p 3 or R/A Pru p 3 or with PBS (control) every week in a total of eight times. One week after the final SCIT (day 92), the mice received intraperitoneal challenge with 100 *μ*g of Pru p 3. Thirty minutes after the challenge, body temperatures were measured. Moreover, physical and behavioural symptoms were assessed according to a scoring system [16]: 0: no symptoms; 1: scratching and rubbing around the nose and head; 2: puffiness around the eyes and mouth, diarrhoea, reduced activity, and/or decreased activity with increased respiratory rate; 3: wheezing, laboured respiration, and cyanosis around the mouth and the tail; 4: no activity after prodding or tremor and convulsion; and 5: death. Mice were euthanized, and the blood and spleens were harvested for immunological assays. Animal experiments were conducted according to the international standards of animal welfare and approved by the Animal Experimentation Ethics Committee of BIONAND, Malaga, Spain.

### 2.4. Detection of Pru p 3-Specific Antibodies and Its Producing Cells

The levels of Pru p 3-specific IgE, IgG1, and IgG2a antibodies in the sera were measured by ELISA, whereas the numbers of Pru p 3-specific IgE, IgG1, and IgG2a antibody-producing cells in splenocytes were measured by ELISPOT as previously described [13, 14].

### 2.5. Pru p 3-Specific Splenocyte Proliferation and Cytokine Production Assay

The spleens were harvested from the mice after the challenge with Pru p 3. Splenocytes were prepared, stained with carboxyfluorescein succinimidyl ester (CFSE), and cultured in the presence of 25 *μ*g of Pru p 3 subsequently [13, 14].

After 72 h, culture supernatants were collected for cytokine analysis (IFN-*γ*, IL-4, IL-10, and IL-13) by ELISA (all from BD Pharmingen, San Diego, CA, USA). In order to assess splenocyte proliferative responses, after 96 hours of the culture, the cells were stained with specific fluorochrome-conjugated mAbs, anti-CD4-PE, and anti-CD8-PE-Cy7A (BD Pharmingen), phenotyped with a BD™ FACSCanto II flow cytometer and analyzed using FlowJo® software (Tree Star Inc., USA). Results were expressed as proliferation index (PI) for CD4^+^ or CD8^+^ measured as the ratio: % CD4^+^CFSE^dim^ or % CD8^+^CFSE^dim^ in stimulated sample/% CD4^+^CFSE^dim^ or % CD8^+^CFSE^dim^ in nonstimulated sample and considered positive when the ration was higher than 2 [13, 14].

### 2.6. Statistical Analysis

Data were presented as individual values and mean with SD values. Quantitative nonrelated variables were analyzed using the Mann–Whitney *U* test. *p* values lower than 0.05 were considered statistically significant.

## 3. Results

### 3.1. Pru p 3 and R/A Pru p 3 Retain Stability in a Cold Storage Condition

**Figure 1:**
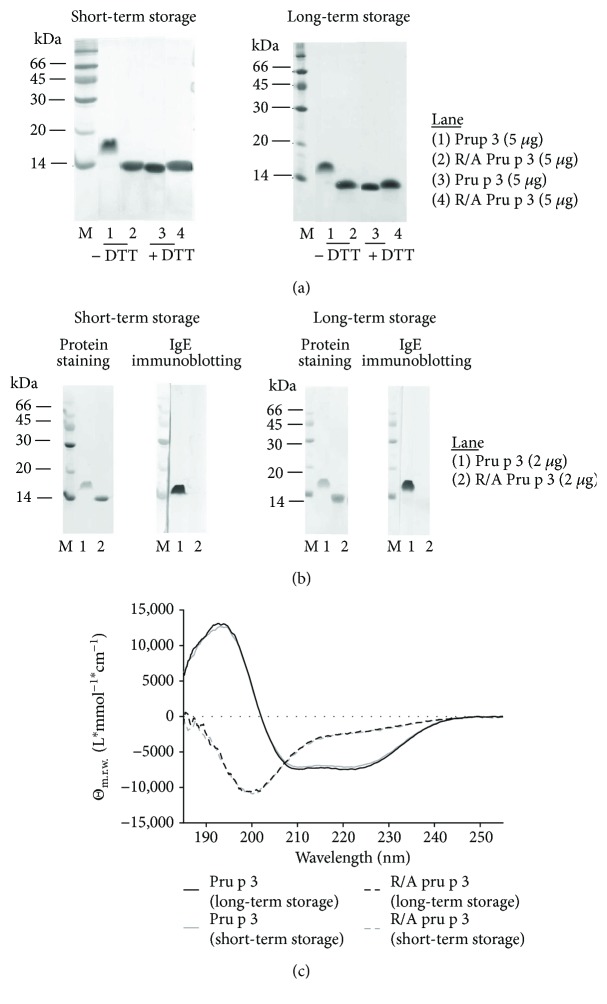
R/A Pru p 3 retained stability after long-term storage. (a) SDS-PAGE and Coomassie staining Pru p 3 and R/A Pru p 3. Left column: samples stored at 4°C for 1 week. Right column: samples stored at 4°C for 14 months. (b) Western blotting using serum from a peach-allergic patient. Left column: samples stored at 4°C for 1 week. Right column: samples stored at 4°C for 14 months. (c) Circular dichroism spectra of Pru p 3 and R/A Pru p 3. MW: molecular weight marker (kDa).

A Pru p 3 folding variant was generated by disruption of disulphide bonds of Pru p 3 upon reduction and alkylation and dialyzed against PBS for *in vivo* application. In nonreducing condition of SDS-PAGE analysis, apparent molecular mass of Pru p 3 and R/A Pru p 3 was approximately 17 kDa and 12 kDa, respectively (Figure 1(a): left column). The reduced molecular mass of R/A Pru p 3 in the nonreducing condition is due to its unfolded structure, since both Pru p 3 and R/A Pru p 3 showed a similar molecular mass of 12 kDa in reducing condition (Figure 1(a)). IgE immunoblotting using serum from a peach-allergic patient reacting to Pru p 3 showed a positive band of Pru p 3, but not of R/A Pru p 3, confirming the hypoallergenic property of R/A Pru p 3 (Figure 1(b)).

To assess the stability of R/A Pru p 3 in a storage condition, we stored Pru p 3 samples in PBS at 4°C and applied it to SDS-PAGE. However, disulphide-mediated aggregation and degradation were not detected in both of Pru p 3 and R/A Pru p 3 in a Coomassie stained gel even after long-term storage at 4°C for 14 months (Figure 1(a): right column). SDS-PAGE and immunoblotting showed similar results of freshly prepared short-term and long-term stored Pru p 3 samples (Figures 1(a) and 1(b)). Chromatogram in a gel filtration analysis confirmed that Pru p 3 and R/A Pru p 3 retained monomeric property after long-term storage at 4°C for 14 months (Fig.  S1). The CD spectrum of R/A Pru p 3 verified a typically unfolded protein, whereas Pru p 3 showed two minima at 208 nm and 222 nm indicating an *α*-helical folded structure (Figure 1(c)). The results suggest that R/A Pru p 3 is stable, at least in PBS at 4°C for 14 months, and can be used for immunological analyses.

### 3.2. SCIT with Pru p 3, but Not R/A Pru p 3, Suppresses the Development of Anaphylaxis

**Figure 2:**
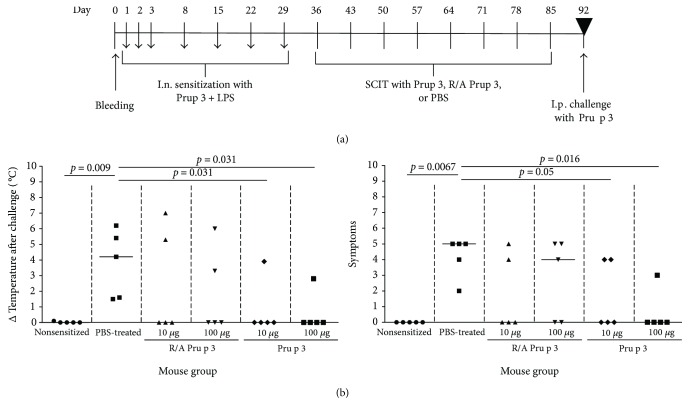
SCIT with Pru p 3, but not with R/A Pru p 3, suppressed development of anaphylactic reaction. (a) Immunization schedule. BALB/c mice were sensitized with Pru p 3 plus lipopolysaccharide (LPS) intranasally three times in the first week and subsequently at one-week interval four times with a total of 7 doses. One week after the final sensitization, mice received SCIT with 10 or 100 *μ*g of Pru p 3 or R/A Pru p 3 at one-week interval in a total of eight times. One week after the final SCIT, the mice received intraperitoneal challenge with Pru p 3. (b) Left: dots represent drop of body core temperature after the challenge with Pru p 3 (*x*-axis). Right: clinical score according to a scoring system: 0: no symptoms; 1: scratching and rubbing around the nose and head; 2: puffiness around the eyes and mouth, diarrhoea, “pilar erecti,” reduced activity, and/or decreased activity with increased respiratory rate; 3: wheezing, laboured respiration, and cyanosis around the mouth and the tail; 4: no activity after prodding or tremor and convulsion; and 5: death. Symbols represent individual mice in each group.

To assess the therapeutic efficacy of Pru p 3 and R/A Pru p 3 in SCIT, BALB/c mice were sensitized with Pru p 3 and received SCIT with 10 or 100 *μ*g of either of Pru p 3 samples or PBS (see immunization protocol in Figure 2(a)). Upon the challenge with Pru p 3, the anaphylactic control group receiving subcutaneous injection only with PBS showed drop of body temperature, an anaphylactic symptom. When compared to the PBS-treated mice, only SCIT-treated mice with Pru p 3, both 10 and 100 *μ*g, suppressed the drop of body temperature significantly and blocked the appearance of anaphylactic symptoms (Figure 2(b)). SCIT with 100 *μ*g of Pru p 3 suppressed the drop of body temperature in 4 out of 5 mice significantly, whereas SCIT with 100 *μ*g of R/A Pru p 3 suppressed it only in 2 out of 5 mice. SCIT with 10 *μ*g of Pru p 3 or R/A Pru p 3 suppressed the drop of body temperature in 3 out of 5 mice. However, in the group of SCIT with 10 or 100 *μ*g of R/A Pru p 3, 2 out of 5 mice showed strong drop of body temperature. The results suggest that SCIT was efficient with a high dose of Pru p 3 to suppress development of peach allergen-induced anaphylaxis significantly.

### 3.3. SCIT with Pru p 3, but Not with R/A Pru p 3, Induces Th1-Associated Antibody Production

**Figure 3:**
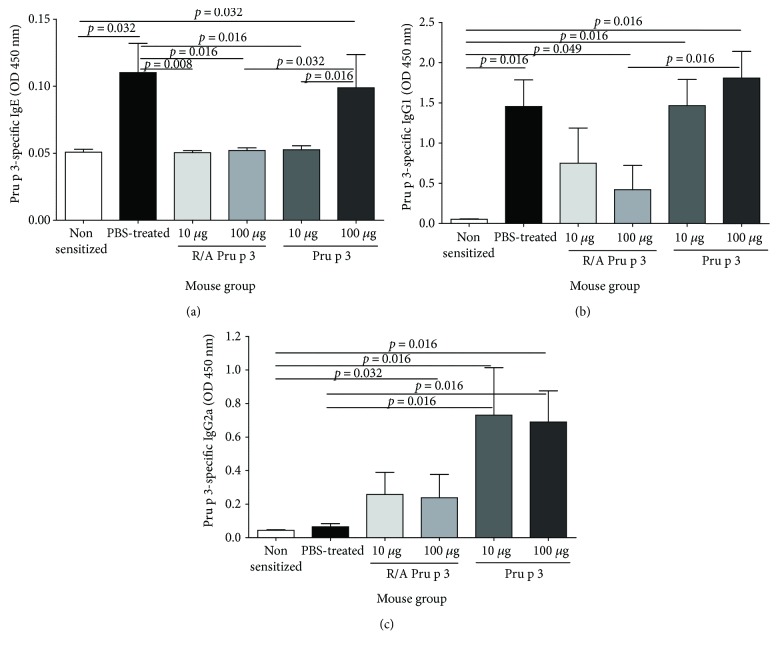
SCIT with Pru p 3, but not with R/A Pru p 3, induced production of Th1-type IgG Abs. After the sensitization with Pru p 3, BALB/c mice received SCIT with 10 or 100 *μ*g of Pru p 3 or R/A Pru p 3 and challenged with Pru p 3. The serum levels of (a) Pru p 3-specific IgE, (b) IgG1, and (c) IgG2a Abs were measured by ELISA.

To obtain an insight into the different efficacies of Pru p 3 and R/A Pru p 3 in SCIT, we assessed antibody production in Pru p 3-sensitized and SCIT-treated mice, Pru p 3-sensitized and PBS-treated group, or nonsensitized and nontreated mice, upon the allergen challenge. Serum levels of Pru p 3-specific IgE, IgG1, and IgG2a antibodies (sIgE, sIgG1, and sIgG2a Abs) in the animals were measured by ELISA, whereas the numbers of cell producing sIgE, sIgG1, and sIgG2a Abs in the spleens were determined by ELISPOT. ELISA analysis showed that SCIT with 100 *μ*g of Pru p 3 retained the levels of sIgE Abs, whereas other treatments significantly reduced IgE production, when compared to the anaphylactic control (Figure 3(a)). The levels of IgG1 Abs were comparable among groups of SCIT-treated mice with 10 or 100 *μ*g of Pru p 3 and PBS-treated mice (Figures 3(b) and 3(c)). SCIT with 10 *μ*g or 100 *μ*g of R/A Pru p 3 induced only a trend to reduce the levels of sIgG1 Abs and to enhance the levels of sIgG2a Abs (Figures 3(b) and 3(c)).

**Figure 4:**
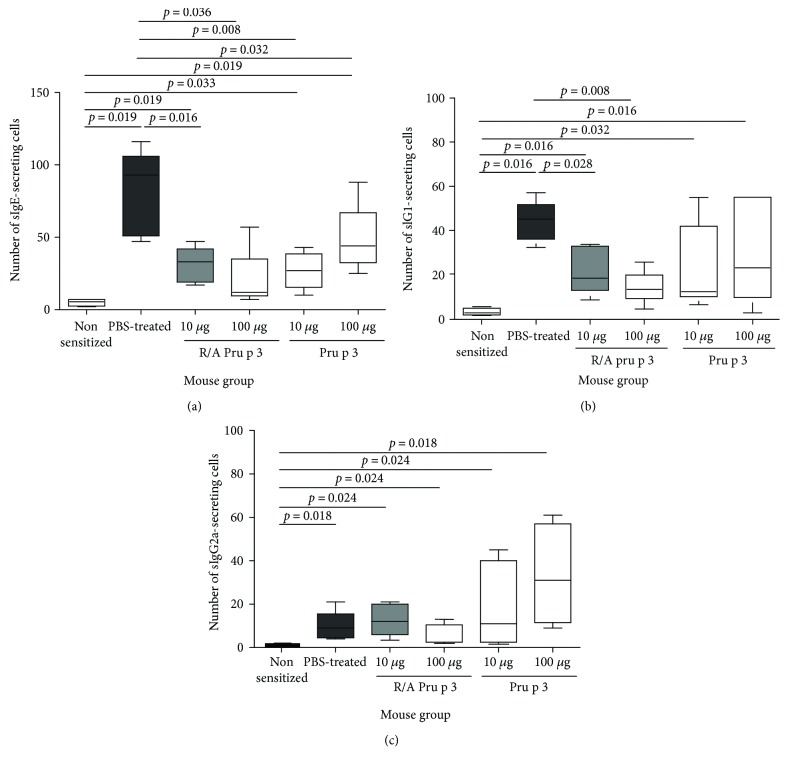
SCIT with Pru p 3, but not with R/A Pru p 3, increased the number of Th1-type IgG Ab-producing cells in the spleen. After the sensitization with Pru p 3, BALB/c mice received SCIT with 10 or 100 *μ*g of Pru p 3 or R/A Pru p 3 and challenged with Pru p 3. The numbers of (a) Pru p 3-specific IgE, (b) IgG1, and (c) IgG2a-producing cells in the spleens were measured by ELISPOT.

ELISPOT analysis showed that all SCIT treatments significantly reduced the number of sIgE-producing cells, although the suppression by 100 *μ*g of Pru p 3 was moderate (Figure 4(a)). SCIT with 10 *μ*g or 100 *μ*g of Pru p 3 did not reduce the number of sIgG1-producing cells, but tended to increase the number of sIgG2a-producing cells (Figures 4(b) and 4(c)). SCIT with 10 *μ*g or 100 *μ*g of R/A Pru p 3 reduced the number of sIgG1-producing cells, but did not influence the number of sIgG2a-producing cells (Figures 4(b) and 4(c)). IgG1 and IgG2a are the Th2- and Th1-associated IgG subclasses, respectively. The ELISA and ELISPOT analyses suggest that (i) SCIT with a high dose of Pru p 3 did not alter levels of Th2-associated antibody production, but enhanced Th1-associated antibody production and (ii) SCIT with a high dose of R/A Pru p 3 suppressed Th2-associated antibody production, but induced Th1-associated antibody production only moderately. SCIT with 10 *μ*g of Pru p 3 or R/A Pru p 3 induced a similar trend in induction of Pru p 3-specific IgG antibodies as observed upon SCIT treatment with a high dose of the respective molecule.

### 3.4. SCIT with Pru p 3, but Not R/A Pru p 3, Suppresses Pru p 3-Specific T-Cell Proliferation

**Figure 5:**
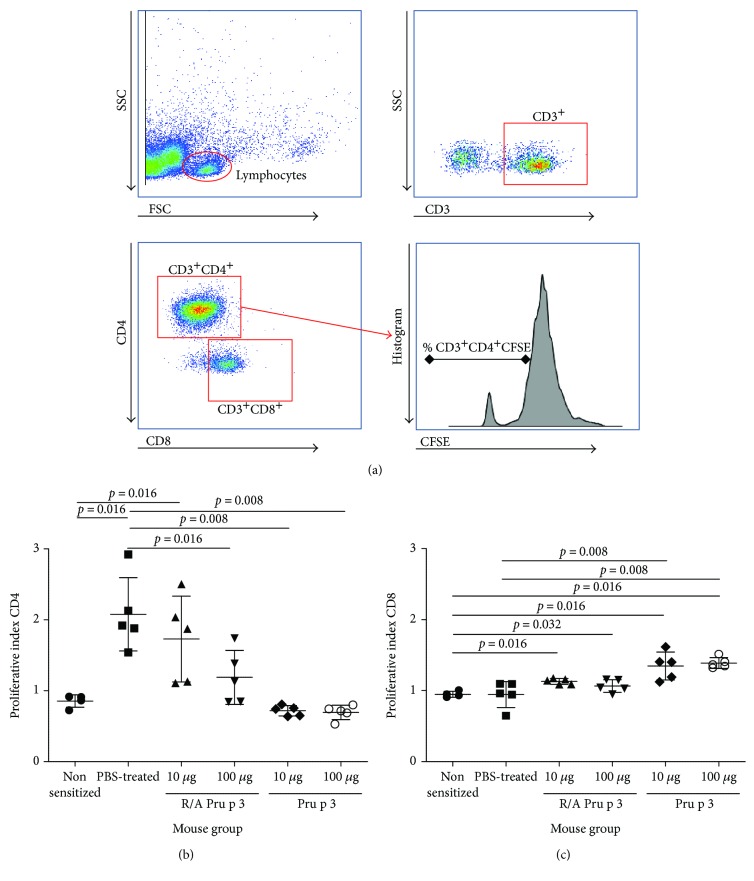
SCIT with Pru p 3 or R/A Pru p 3 suppressed allergen-specific CD4^+^ T-cell proliferation. After the sensitization with Pru p 3, BALB/c mice received SCIT with 10 or 100 *μ*g of Pru p 3 or R/A Pru p 3 and challenged with Pru p 3. Splenocytes from the mice were stained with CSFE and cultured in the presence of Pru p 3 for 96 hours. (a) Gating strategy in flow cytometry analysis for proliferation of (b) CD4^+^ T-cells and (c) CD8^+^ T-cells in the cultured splenocytes.

To assess the effect of SCIT on Pru p 3-specific T-cell responses, *in vitro* antigen recall assay was performed using splenocytes from SCIT-treated or PBS-treated mice. By flow cytometric analysis based on the dilution of CFSE, Pru p 3-specific proliferation of CD4^+^ T-cells, but not CD8^+^ T-cells, was detected in culture of splenocytes from PBS-treated mice (Figures 5(a)–5(c)). Remarkably, splenic CD4^+^ T-cells from SCIT-treated mice with 10 or 100 *μ*g of Pru p 3 did not show detectable levels of proliferation, whereas those from SCIT-treated mice with 10 or 100 *μ*g of R/A Pru p 3 tended to show substantial but lower levels of proliferation when compared to the anaphylactic control. The results suggest that SCIT with Pru p 3 possesses a better suppressive effect on expansion of Pru p 3-specific CD4^+^ T-cells than R/A Pru p 3.

**Figure 6:**
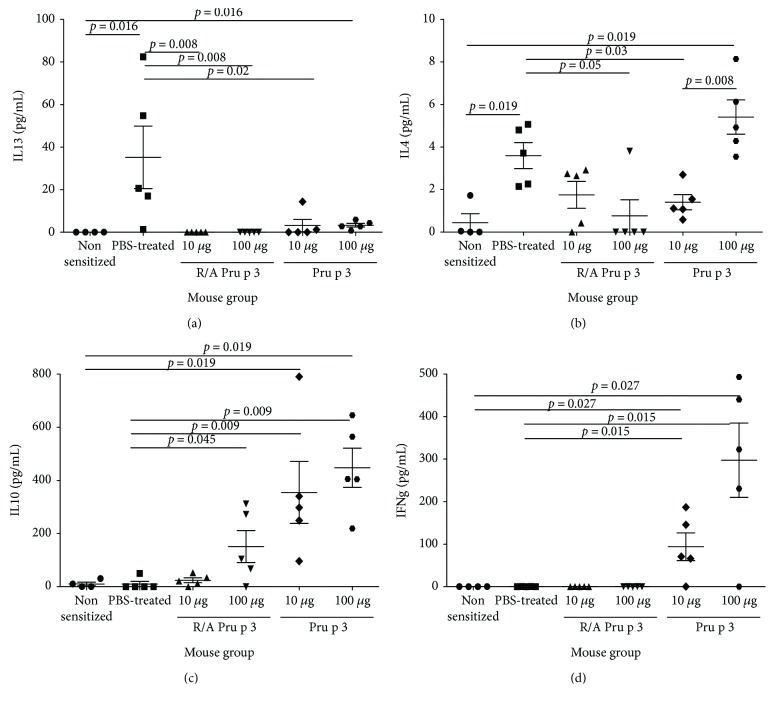
SCIT with Pru p 3 or R/A Pru p 3 suppressed cytokine production in allergen-specific CD4^+^ T-cells. Splenocytes from the mice were cultured in the presence of Pru p 3 for 72 hours. The concentrations of (a) IL-13, (b) IL-4, (c) IL-10, and (d) IFN-*γ* in the culture supernatants were measured by ELISA.

Next, cytokine concentrations in the culture supernatant of Pru p 3-stimulated splenocytes were measured to see the suppressive effect of SCIT with Pru p 3 or R/A Pru p 3 on cytokine responses. Splenocytes from PBS-treated mice produced Th2 cytokines, that is, a high level of IL-13 (Figure 6(a)), and a marginal level of IL-4 (Figure 6(b)), but not detectable levels of a Th1-type cytokine IFN-*γ* and an immunosuppressive cytokine IL-10 (Figures 6(c) and 6(d)). When compared to the PBS-treated control, splenocytes from SCIT-treated mice with 10 or 100 *μ*g of Pru p 3 reduced IL-13 secretion, whereas IFN-*γ* and IL-10 are produced at high levels. Splenocytes from SCIT-treated mice with 10 or 100 *μ*g of R/A Pru p 3 also reduced IL-13 levels, but only those from animals treated with the high dose of R/A Pru p 3 produced detectable levels of IL-10, but not of IFN-*γ*. The results suggest that only SCIT with a high dose of Pru p 3 induces both IFN-*γ*-producing Th1 cells and IL-10-producing regulatory cells.

## 4. Discussion

The use of hypoallergenic variants in AIT has been discussed to improve the safety and efficacy of the therapy. It has been thought that hypoallergens can be applied in higher doses due to reduced allergenicity and therefore provide a safer and more effective treatment than the respective wild-type allergens. However, in the present study, we found that Pru p 3, the wild-type peach allergen, possesses a better therapeutic efficacy than R/A Pru p 3, a hypoallergenic folding variant, in SCIT to suppress development of anaphylaxis.

*In vitro* recall antigen stimulation assay showed that SCIT with Pru p 3, but not with R/A Pru p 3, induced Th1 cells predominantly and suppressed expansion of Pru p 3-specific Th2 cells. IFN-*γ*, a Th1 cytokine, is capable of downregulating allergic responses via (i) direct inhibition of Th2 cell proliferation, (ii) potentiating IL-12 production, which favors differentiation of Th1 cells, (iii) inducing apoptosis via Fas/Fas-L pathway, and (iv) reduction in the number of mast cells [12, 17, 18]. Furthermore, in addition to the Th1-biased immune response, SCIT with Pru p 3 induced higher levels of IL-10-producing cells than SCIT with R/A Pru p 3. IL-10 impairs proliferation and cytokine production of effector T-cells, IgE production of B-cells, and activation of Fc*ε*RI-engaged mast cells [19]. Taken together, our result suggests that the increases of IFN-*γ* and IL-10 are markers related with the observed therapeutic efficacy.

SCIT-treated mice with 100 *μ*g of Pru p 3 increased Pru p 3-specific IgE levels, compared to those with 10 *μ*g of Pru p 3 or 10 or 100 *μ*g of Pru p 3. It is worth noting that transient early increases in serum allergen-specific IgE antibody levels have been often observed in both sublingual and subcutaneous immunotherapy [1, 12]. These increases are not accompanied by untoward side effects, and it has been suggested that early Th2 priming by high allergen exposure might be important for successful immunotherapy [1, 12]. Reduction in allergen-specific IgE levels has been observed after prolonged subcutaneous immunotherapy over several years [12], although mechanism for the reduction is still not well elucidated.

The differential effects of Pru p 3 and R/A Pru p 3 in SCIT could be attributed to differences in the antigenicity of these molecules. SCIT with Pru p 3, but not with R/A Pru p 3, also induced significant increase of Pru p 3-specific IgG Abs. This is consistent with our previous study showing that R/A Pru p 3 loses antigenicity almost completely [11]. Several studies have suggested that in addition to induction of regulatory cells and/or Th1 cells, the rise in blocking IgG Abs induced by therapeutic allergens is critical for the efficacy of AIT [11, 19–22]. Blocking IgG Abs antagonize allergen recognition by IgE Abs and/or induce inhibitory signaling via Fc*_γ_*RIIb to suppress the cascade of allergic reactions. In addition, IgG Abs are capable of promoting antigen presentation by dendritic cells [23]. The association of allergen with IgG bound to Fc*_γ_*RI on the surface of dendritic cells induces endocytosis of allergen to deliver it into antigen presentation pathway for T-cell stimulation. Stimulation of CD4^+^ T-cells at high antigen concentration by dendritic cells tends to induce Th1 cell differentiation [24]. Our results suggest that both T-cell immunogenicity and antigenicity need to be retained in hypoallergenic variants in order to induce its therapeutic efficacy in AIT.

One could postulate that the better efficacy of Pru p 3 is associated with the stability of the protein. Pru p 3 was one of the allergens highlighted in the FAST (Food Allergy Specific ImmunoTherapy) project targeting persistent and severe allergy to fish and peach. The FAST project was aimed at developing safe and effective SCIT for food allergies using hypoallergens [25, 26]. However, at the initial stage of the project, the further clinical development of hypoallergenic unfolded Pru p 3 was skipped, because recombinant Pru p 3 substituted at cysteines with serines, or subjected to reduction and alkylation, displayed weak stability in several storage conditions [26]. In contrast to the FAST project, we observed that R/A Pru p 3 retains the stability in PBS at 4°C at least for 14 months. Therefore, it is less likely that the lower therapeutic efficacy of R/A Pru p 3 is due to protein degradation, or aggregation, which often occurs to other unfolded proteins during storages. However, we cannot exclude that defolding of Pru p 3 results in more efficient degradation *in vivo*, and thereby, R/A Pru p 3 cannot manifest the therapeutic efficacy to suppress allergic responses in SCIT. It is well known that proteins and peptides are degraded by proteases at the subcutaneous injection sites [27]. We previously observed that R/A Pru p 3 was easily digested to peptides by proteases due to its unfolded structure [11]. Therefore, R/A Pru p 3 would not be stable when it is injected subcutaneously.

In summary, we demonstrated that a hypoallergenic unfolded variant of Pru p 3 is not likely a suitable component in SCIT to treat peach allergy. The lower therapeutic efficacy of R/A Pru p 3 could be due to its low stability in SCIT. Our findings contribute to improving design strategies for hypoallergens with high clinical efficacy to treat food allergy in AIT.

## Abbreviations

nsLTP: Nonspecific lipid transfer protein
R/A: Reduced and alkylated
SCIT: Subcutaneous immunotherapy.

## Supplementary Materials

Supplementary Materials Fig. S1: analytical size exclusion chromatography of Pru p 3 samples. (A) Pru p 3 (red line) or R/A Pru p 3 (brown line) or (B) a standard mixture was applied to Sephadex 75 pg HR30/10 column. Based on the standard, molecular weights of Pru p 3 and R/A Pru p 3 were estimated at 12.25 and 15.247 kDa, respectively. The results suggest that both Pru p 3 samples retained monomeric form. Higher molecule weight of R/A Pru p 3 would be explained by its unforded property.
