# Supplementary material for: Immunotherapy with Native Molecule rather than Hypoallergenic Variant of Pru p 3, the Major Peach Allergen, Shows Beneficial Effects in Mice
Source: J Immunol Res. 2018 Jun 13;2018:3479185. doi: 10.1155/2018/3479185 (PMC6020533; doi:10.1155/2018/3479185)
Supplement: Supplementary Materials — Fig. S1: analytical size exclusion chromatography of Pru p 3 samples. (A) Pru p 3 (red line) or R/A Pru p 3 (brown line) or (B) a standard mixture was applied to Sephadex 75 pg HR30/10 column. Based on the standard, molecular weights of Pru p 3 and R/A Pru p 3 were estimated at 12.25 and 15.247 kDa, respectively. The results suggest that both Pru p 3 samples retained monomeric form. Higher molecule weight of R/A Pru p 3 would be explained by its unforded property. [file 3479185.f1.pdf]

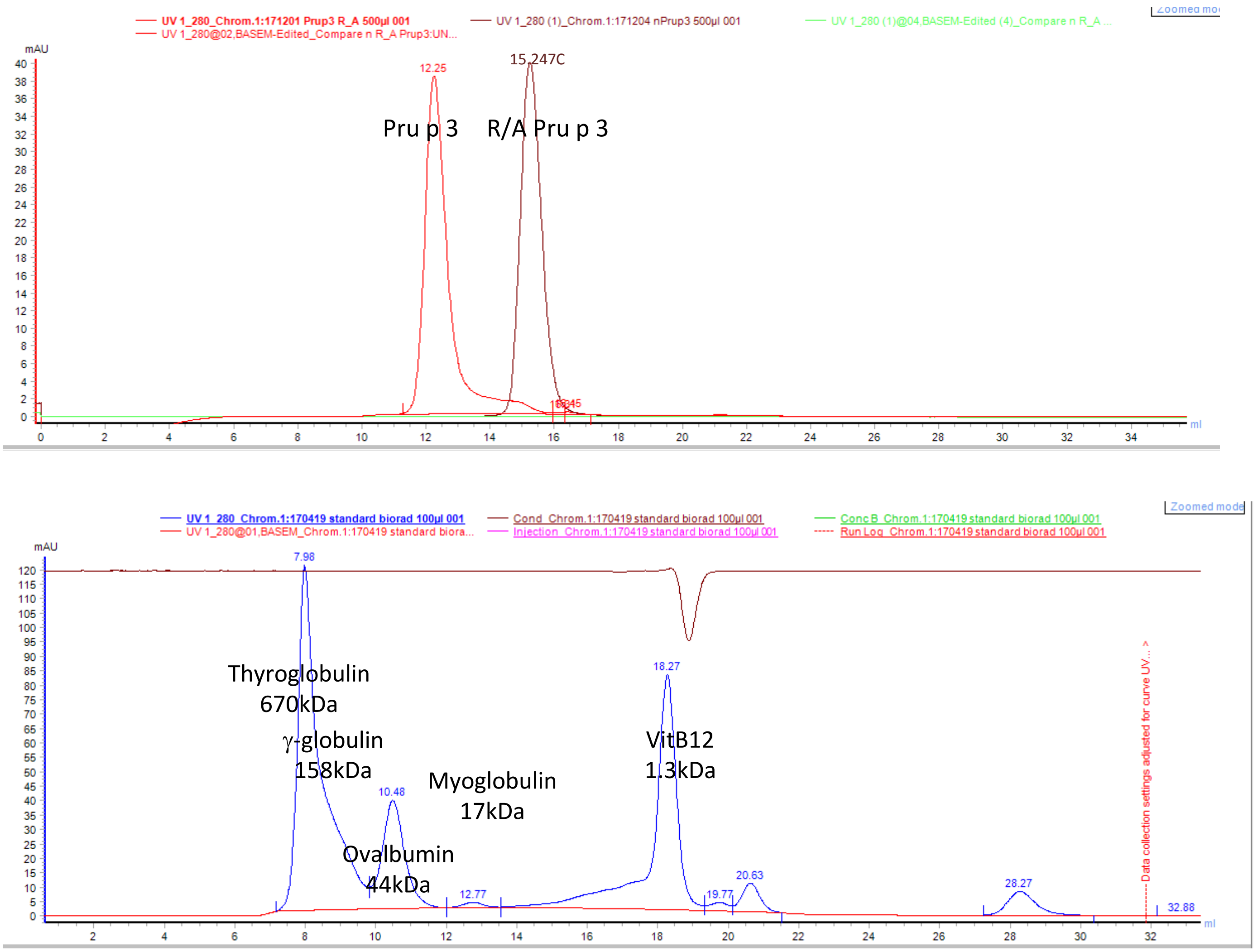

Fig. S1. Analytical size exclusion chromatography of Pru p 3 samples. (A) Pru p 3 (red line), or R/A Pru p 3 (brown line), or (B) a standard mixture was applied to Sephadex 75pg HR30/10 column. Based on the standard, molecular weights of Pru p 3 and R/A Pru p 3 were estimated at 12.25 and 15.247 kDa, respectively. The result suggest that both Pru p 3 samples retained monomeric form. Higher molecule weight of R/A Pru p 3 would be explained by its unforded property.
